# N-Doped carbon nanoparticles on highly porous carbon nanofiber electrodes for sodium ion batteries†

**DOI:** 10.1039/d3ra00635b

**Published:** 2023-03-09

**Authors:** Meltem Yanilmaz, Bülin Atıcı, Jiadeng Zhu, Ozan Toprakci, Juran Kim

**Affiliations:** a Nano-Science and Nano-Engineering Program, Graduate School of Science, Engineering and Technology, Istanbul Technical University Istanbul 34469 Turkey yanilmaz@itu.edu.tr; b Textile Engineering, Istanbul Technical University Istanbul 34469 Turkey; c Chemical Sciences Division, Oak Ridge National Laboratory Oak Ridge TN 37831 USA; d Department of Polymer Materials Engineering, Yalova University 77200 Yalova Turkey; e Advanced Textile R&D Department, Korea Institute of Industrial Technology (KITECH) Ansan 15588 Korea jkim0106@kitech.re.kr

## Abstract

Nitrogen doped carbon nanoparticles on highly porous carbon nanofiber electrodes were successfully synthesized *via* combining centrifugal spinning, chemical polymerization of pyrrole and a two-step heat treatment. Nanoparticle-on-nanofiber morphology with highly porous carbon nanotube like channels were observed from SEM and TEM images. Nitrogen doped carbon nanoparticles on highly porous carbon nanofiber (N-PCNF) electrodes exhibited excellent cycling and C-rate performance with a high reversible capacity of around 280 mA h g^−1^ in sodium ion batteries. Moreover, at 1000 mA g^−1^, a high reversible capacity of 172 mA h g^−1^ was observed after 300 cycles. The superior electrochemical properties were attributed to a highly porous structure with enlarged *d*-spacings, enriched defects and active sites due to nitrogen doping. The electrochemical results prove that N-PCNF electrodes are promising electrode materials for high performance sodium ion batteries.

## Abstract 2

Nitrogen doped carbon nanoparticles on highly porous carbon nanofiber electrodes were successfully synthesized *via* combining centrifugal spinning, chemical polymerization of pyrrole and a two-step heat treatment.

## Graphical abstract

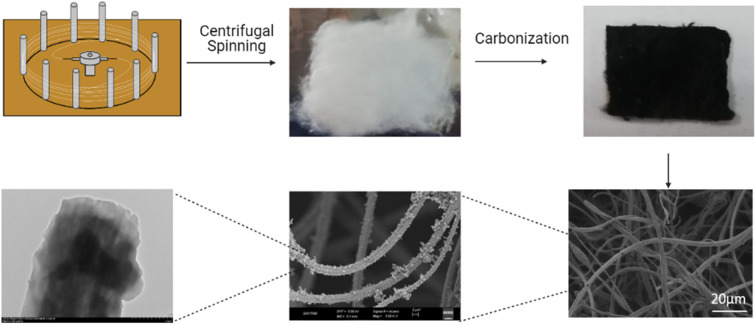

## 1. Introduction

Lithium ion batteries (LIBs) have been the most widely used rechargeable batteries in portable electronics. However, limited sources and the high price of lithium metal limit the application of LIBs in power grids and electric vehicles. Considering the abundant sodium sources, sodium ion batteries (SIBs) have been presented as an alternative to LIBs. However, low energy density and the poor cycle life of SIBs need to be addressed for further development of SIBs.^1–8^

Carbon materials have been reported as electrodes for SIBs owing to their good electrical conductivity, cost effectiveness, and excellent chemical and physical stability, as well as their high porosity and good cycling performance.^9–12^ Moreover, disordered carbons with larger interlayer space are beneficial for Na^+^ insertion and extraction. Fabricating porous carbon as well as N-doping have been studied for improving the capacity and cycling performance of carbon anodes. Porous carbon nanofibers have the merits of high conductivity, excellent flexibility, a short ion diffusion pathway, good stress tolerance and a large surface area to volume ratio.^13,14^ N doping creates defects and improves electronic conductivity, thus electrochemical performance is improved by providing more sites for Na^+^ storage.^15–17^

Polyacrylonitrile (PAN) has been commonly used as carbon precursor owing to its high carbon yield and good mechanical properties.^18–22^ Production of high performance electrode materials *via* low cost and facile approach is vital to use sodium ion batteries in practical applications.^3,9,23^ Morphology, porosity and pore structure, affect the electrochemical properties of carbon materials and nanofiber production technique influence the porosity and pore structure. Centrifugal spinning allows fast and cost-effective production of nanofibers and centrifugally spun nanofibers show higher porosity compared to electrospun nanofibers. Furthermore, porosity and pore size could be easily adjusted in this technique.^19,24^ Polymethylmethacrylate (PMMA) and polystyrene (PS) are the most commonly used polymers to create pore structure in carbon fibers because of their low carbon yield. These polymers decompose and create pores during high temperature carbonization processes.^3,25,26^ The novel morphology of N-doped carbon nanoparticles on porous carbon nanofibers obtained from polypyrrole (PPy) coated centrifugally spun PAN/PMMA and PAN/PS nanofibers have not been studied yet. Herein, two types of blend nanofibers, PAN/PMMA and PAN/PS were centrifugally spun as precursors to prepare highly porous carbon nanofiber electrodes for sodium ion batteries for the first time. The effect of low carbon yield polymer type (PMMA and PS) and content of these polymers on the morphology of carbon nanofibers were investigated. Furthermore, N-doped carbon nanoparticles on highly porous carbon nanofiber electrodes were synthesized *via* combining centrifugal spinning, chemical polymerization of pyrrole and two step heat treatment. N-PCNF electrodes delivered high reversible capacity with excellent cycling performance owing to N-doping and highly porous structure with enlarged interlayer spacings. Considering low cost and facile production approach and excellent electrochemical properties, nitrogen doped carbon nanoparticles on highly porous carbon nanofiber electrodes are promising electrode materials for sodium ion batteries.

## 2. Experimental

### 2.1 Chemicals

Polyacrylonitrile (PAN, *M*_w_ = 150 000), polymethylmethacrylate (PMMA, *M*_w_ = 120 000), polystyrene (PS, *M*_w_ = 192 000), pyrrole, ammonium per sulfate (APS), hydrochloric acid, ethanol and *N*,*N*-dimethyl formamide (DMF) were supplied from Sigma-Aldrich.

### 2.2 Preparation of N-doped carbon nanoparticles on highly porous carbon nanofibers

PAN/PMMA blend solutions with two different PAN/PMMA weight ratios (1/0.2, 1/0.4) and PAN/PS blend solution with the ratio of 1/0.4 (PAN/PS) were prepared in DMF and labeled as PAN/PMMA1, PAN/PMMA2 and PAN/PS, respectively. These blend solutions were fed into the centrifugal spinning device by a syringe pump. The rotational speed of 4000 rpm, the feeding rate of 60 ml h^−1^, the collector distance of 20 cm, and the nozzle diameter of 0.5 mm was applied in the centrifugal spinning. 10 wt% polymer blend solutions were used in the centrifugal spinning and approximately 5.7 g of nanofibers produced after an hour due to the losses of the solution during production. PAN, PAN/PMMA1, PAN/PMMA2 and PAN/PS were stabilized at 250 °C for 2.5 h in air and carbonized at 800 °C for 2 h in N_2_ and named as CNFs, PCNF1, PCNF2 and PCNF3, respectively. The carbon yield was around 40 wt%. In order to synthesize nitrogen doped carbon nanoparticles on highly porous carbon nanofibers, pyrrole was polymerized on PANPMMA2 and PAN/PS blend nanofibers by using APS as an initiator. In order to further improved the electrochemical performance of PCNFs, PANPMMA2 and PAN/PS blend nanofiber precursors were selected for PPy coating because of the higher porosity and enhanced capacity of PCNFs prepared from PANPMMA2 and PAN/PS blend nanofibers compared to those of CNFs prepared by using PAN and PAN/PMMA1. Pyrrole and APS were dissolved in aqueous hydrochloric acid solution separately. PANPMMA2 and PAN/PS blend nanofibers were immersed in pyrrole solution at 0 °C and then APS solution was added dropwise to pyrrole solution. The molar ratio of pyrrole to APS was 1 : 1 and the polymerization was ended in 12 hours. Polypyrrole (PPy) coated PANPMMA2 and PAN/PS blend nanofibers were washed and dried and then PPy coated PANPMMA2 and PAN/PS blend nanofibers were stabilized at 250 °C for 2.5 h in air and carbonized at 800 °C for 2 h in N_2_ and then named as N-PCNF4 and N-PCNF5 respectively.

### 2.3 Structure characterization

The morphology of nanofibers was investigated by FEI Quanta FEG 250 scanning electron microscope and Hitachi HighTech HT7700 tunneling electron microscope (TEM). The structure of all studied sample was analyzed by using XRD and Raman spectroscopy.

### 2.4 Electrochemical characterization

A slurry of N-PCNFs, carbon black and sodium alginate in deionized water in the weight ratios of 0.8 : 0 : 1 : 0.1 was coated on Cu foil and dried at 60 °C overnight and the electrode weight was approximately 1.5 mg cm^−2^. The electrodes were assembled in CR2032 coin type cells with Na metal and the electrolyte of 1 M NaCIO_4_ in EC : PC (1 : 1 in volume). Galvanostatic charge discharge measurements were performed *via* Neware and Hefa battery test systems at room temperature.

## 3. Results and discussion

### 3.1 Structural characterization

**Fig. 1:**
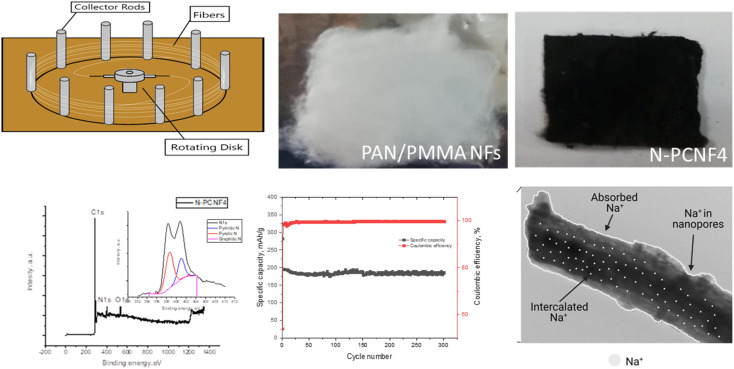
Schematic diagram of the preparation of N-PCNF4.

Schematic diagram of the preparation of nitrogen doped carbon nanoparticle on highly porous carbon nanofibers (N-PCNF) was presented in Fig. 1. Centrifugally spun PAN, PAN/PMMA, PAN/PS were prepared and then pyrrole was polymerized on PAN/PMMA and PAN/PS nanofibers by using APS as oxidant. Polypyrrole coated PAN/PMMA and PAN/PS nanofibers were used as precursors to synthesize nitrogen doped carbon nanoparticles on highly porous carbon nanofibers while PS and PMMA were used as pore generator. 1D porous channels observed from TEM images were fabricated *via* removal of PMMA or PS during heat treatment while PPy was used for N-doping and carbon nanoparticle precursors.

**Fig. 2:**
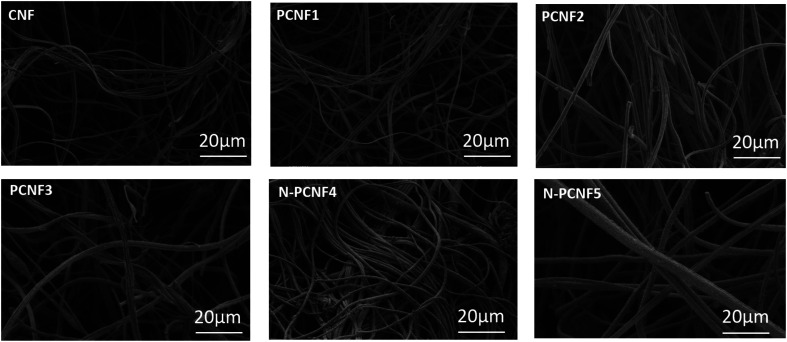
SEM images of CNF, PCNF1, PCNF2, PCNF3, N-PCNF4 and N-PCNF5.

**Fig. 3:**
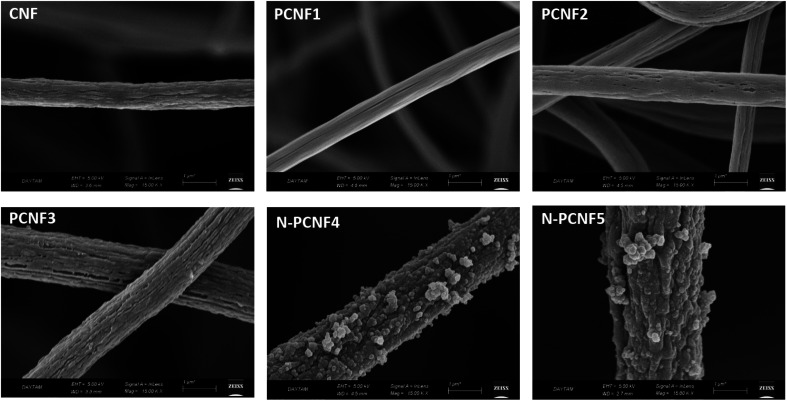
SEM images of CNF, PCNF1, PCNF2, PCNF3, N-PCNF4 and N-PCNF5 with higher magnification.

**Fig. 4:**
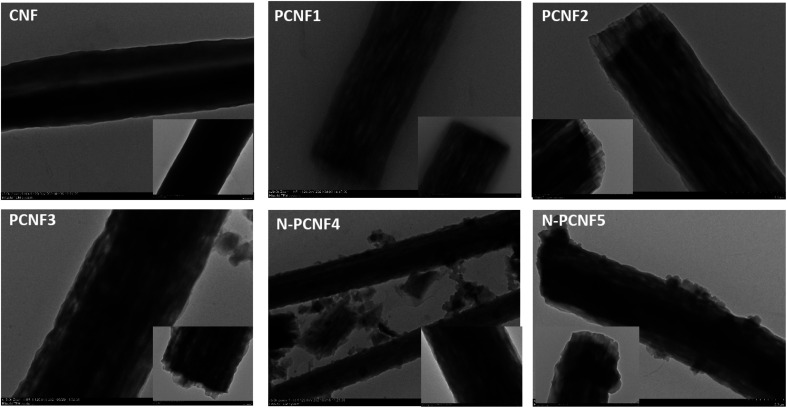
TEM images of CNF, PCNF1, PCNF2, PCNF3, N-PCNF4 and N-PCNF5.

The morphologies of CNFs, PCNFs and N-PCNFs were investigated through SEM and TEM images. SEM images of CNF, PCNF1, PCNF2, PCNF3, N-PCNF4 and N-PCNF5 are presented in Fig. 2. Fibrous structure was observed from all studied carbon nanofibers. In order to evaluate the effect of blend ratio and blend polymer type on the morphology, SEM images with higher magnification are presented in Fig. 3. CNFs had rough surface while PCNF1, PCNF2 and PCNF3 fabricated from PAN/PMMA and PAN/PS nanofibers had pores on fiber surfaces. SEM images of N-PCNF4 and N-PCNF5 show the nanoparticle-on-nanofiber morphology. Moreover, as shown in Fig. 4, TEM images of PCNF1, PCNF2 and PCNF3 depict highly porous structure with tubular structure. PCNFs synthesized from PAN/PMMA and PAN/PS showed similar tubular structure after carbonization without significant difference. The nanoparticle-on-nanofiber morphology with highly porous nanofibers was observed from TEM images of N-PCNF4 and N-PCNF5 as well. SEM and TEM images are consistent with each other and nanoparticle on nanofiber structure was observed from both SEM and TEM images of N-PCNF4 and N-PCNF5. It has been reported that tubular hollow structures can act as a buffer and relieve stresses caused by Na^+^ insertion and extraction during cycling and improve structural stability. Furthermore, larger surface areas and N doping could further improve the capacity and high rate cycling stability.^13^

The surface areas of the CNF, PCNF1, PCNF2, PCNF3, N-PCNF4 and N-PCNF5 were 162, 176, 214, 183, 265, 219 m^2^ g^−1^, respectively, as reported in Table 1. N_2_ adsorption/desorption curves were also presented in Fig. S1.† As compliance with the SEM and TEM images, increasing porosity and N doping led to higher surface areas which is beneficial for Na ion insertion and extraction since large surface area provides larger contact and improved transport of ions and electrons. Increasing surface area with N doping was also observed from N doped carbon framework by Huang *et al.*^27^ and the result was ascribed to disturbed carbon framework and more exposed surface area caused by N doping. Pore volume was also presented in Fig. S1b.† There are micro, meso and macropores in the electrodes. It has been reported that diverse nanoporosity increased the capacity by creating more nanochannels and thus let fast kinetics during Na^+^ intercalation and extraction.^7^

**Table 1:** Specific surface area values of CNFs, PCNFs and N-PCNFs

	Surface area, m^2^ g^−1^
CNF	162
PCNF1	176
PCNF2	214
PCNF3	183
N-PCNF4	265
N-PCNF5	219

**Fig. 5:**
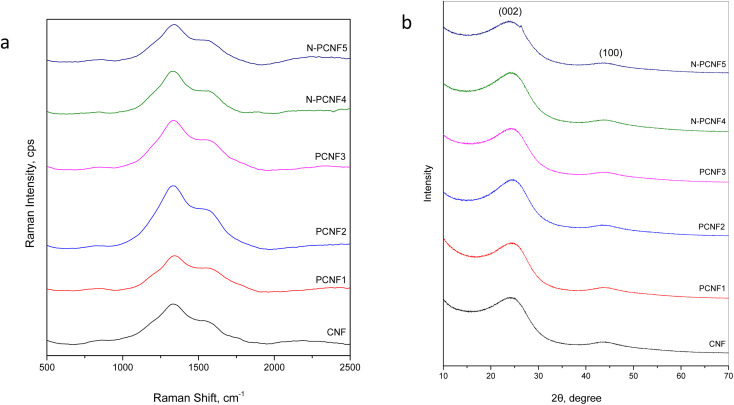
Raman (a) spectra and XRD pattern (b) of CNF, PCNF1, PCNF2, PCNF3, N-PCNF4 and N-PCNF5.

Raman spectra of CNF, PCNF1, PCNF2, PCNF3, N-PCNF4 and N-PCNF5 are exhibited in Fig. 5a. Two peaks at 1340 and 1540 cm^−1^ are ascribed to D band and G bands respectively. The intensity ratio between D and G band (*I*_D_/*I*_G_) is sensitive to carbon microstructure, defects and degree of disorder. The *I*_D_/*I*_G_ values for N-PCNF4 is 1.9 whereas other samples have the ratio of 1.7. The high intensity ratio proves increased disordered structure and defects and it could be ascribed to N doping.^1,15^

XRD patterns of CNF, PCNF1, PCNF2, PCNF3, N-PCNF4 and N-PCNF5 are presented in Fig. 5b. Two broad peaks which are around 25° and 44° can be ascribed to (002) and (100) planes of graphite. The low intensity and broad peaks indicate highly amorphous structure.^1,26,28^ Moreover, interlayer spacings (*d*_(002)_) were found to be 0.36 for CNFs and PCNFs which is larger than that of graphite (0.34 nm) resulting better Na^+^ storage. For Na^+^ storage, highly disordered matrix is beneficial.^16^ The large interlayer spacings of 0.37 are seen for N-PCNF4 and N-PCNF5 which is consistent with Raman spectra and hence proves N doping. Larger interlayer spacing caused by N doping was also reported for pitch derived carbons by Hao *et al.*^29^ Both XRD and Raman spectra confirm larger *d* spacings and enhanced defects which is beneficial for Na^+^ storage properties.

**Fig. 6:**
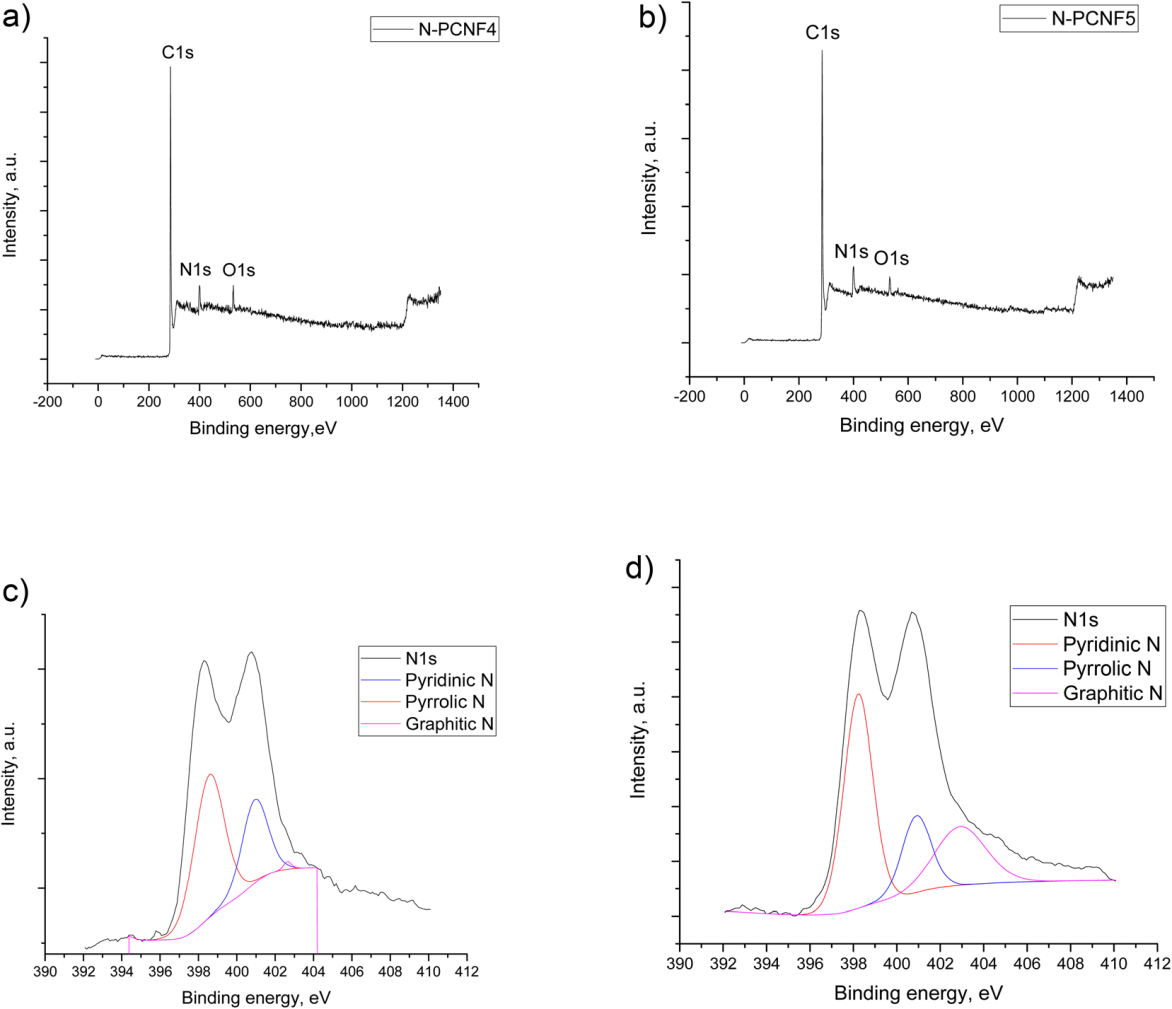
XPS survey (a and b) and N 1s deconvoluted plots (c and d) of N-PCNF4 and N-PCNF5.

To provide further structural analysis, XPS characterization was performed and XPS survey for N-PCNF4 and N-PCNF5 are presented in Fig. 6. Both N-PCNF4 and N-PCNF5 have clear N1s peaks, indicating a high amount of nitrogen doping. Nitrogen contents are 9 and 6%, respectively for N-PCNF4 and N-PCNF5. Moreover, the deconvolution of N1s peaks showed that the compositions of pyridinic and pyrrolic are high which result in more conductive and reversible sites for Na^+^ ion adsorption.^7,30^ The amounts of graphitic-N (402.6 eV), pyrrolic-N (400.8 eV) and pyridinic-N (398.5 eV) are 1, 33, and 66%, respectively for N-PCNF4 whereas those are 26, 20 and 54% respectively for N-PCNF5 which confirms Raman results that suggest higher disordered structure of N-PCNF4.

### 3.2 Electrochemical characterization

**Fig. 7:**
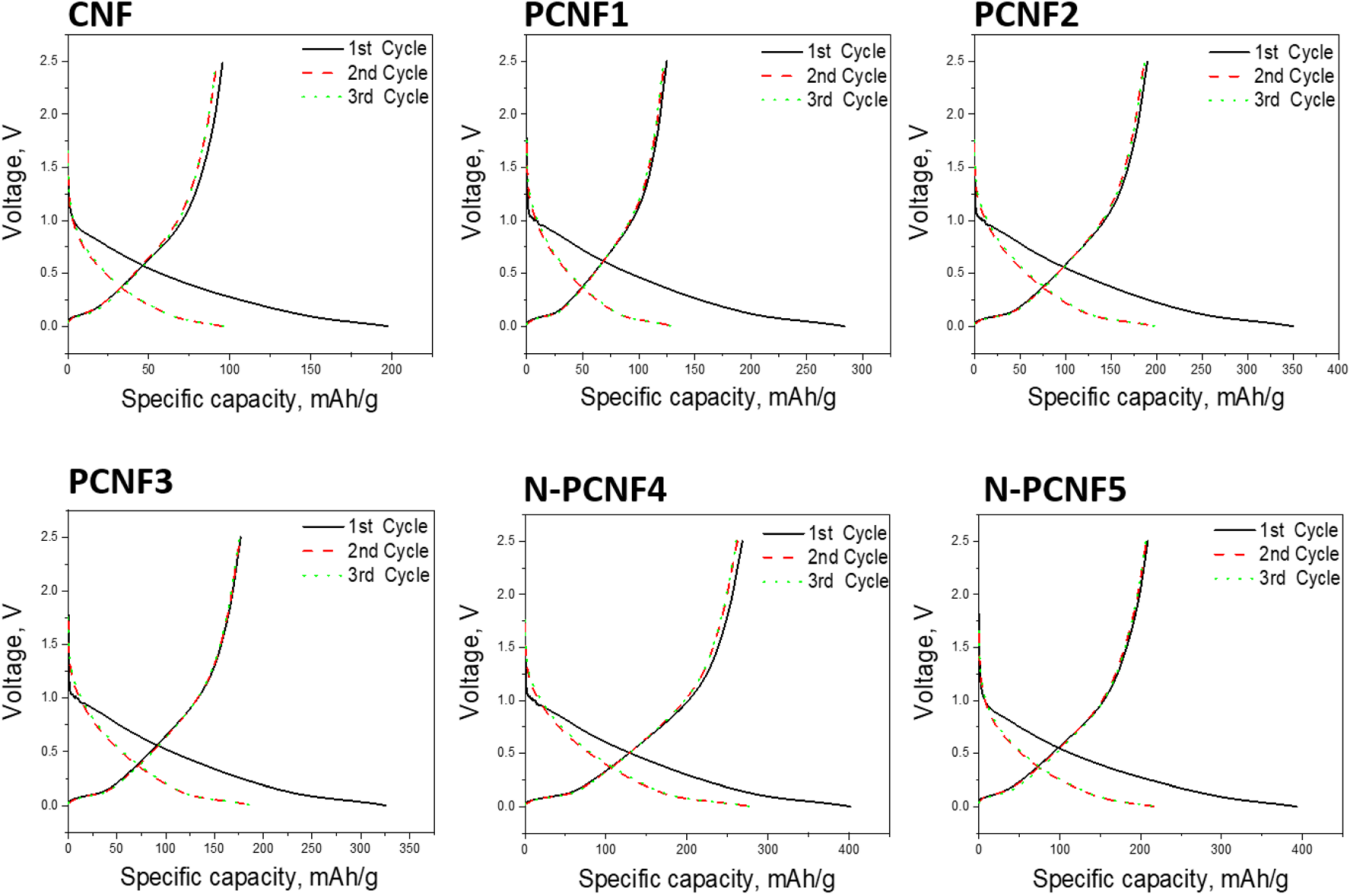
First cycle charge discharge curves for CNF, PCNF1, PCNF2, PCNF3 N-PCNF4 and N-PCNF5.

The sodium storage properties of the electrodes were investigated in 2032 type half cells using sodium as the counter electrode. Fig. 7 shows the discharge and charge curves for the first cycles for all studied electrodes. During the first cycle, the discharge capacities are 197, 284, 350, 325, 402 and 393 mA h g^−1^ respectively for CNF, PCNF1, PCNF2, PCNF3, N-PCNF4 and N-PCNF5 while the charge capacities are 95, 136, 189, 176, 266 and 212 mA h g^−1^ respectively for CNF, PCNF1, PCNF2, PCNF3, N-PCNF4 and N-PCNF5. The large irreversible capacity in the first cycle can be attributed to the electrolyte decomposition and formation of SEI layer.^15^ In the following charge discharge cycles, capacities became stable, indicating reversible intercalation/deintercalation of Na^+^ (Table S1 and Fig. S2†). Moreover, the small plateau and large sloping capacity regions indicate that a large part of the capacity was contributed by adsorption of Na^+^ ions into the nanopores and the reversible redox reaction between Na^+^ ions and functional groups, whereas a minor capacity was shared by intercalation in graphitic nanolayers as reported before for hard carbon included carbon nanofiber electrodes.^7,31^

**Fig. 8:**
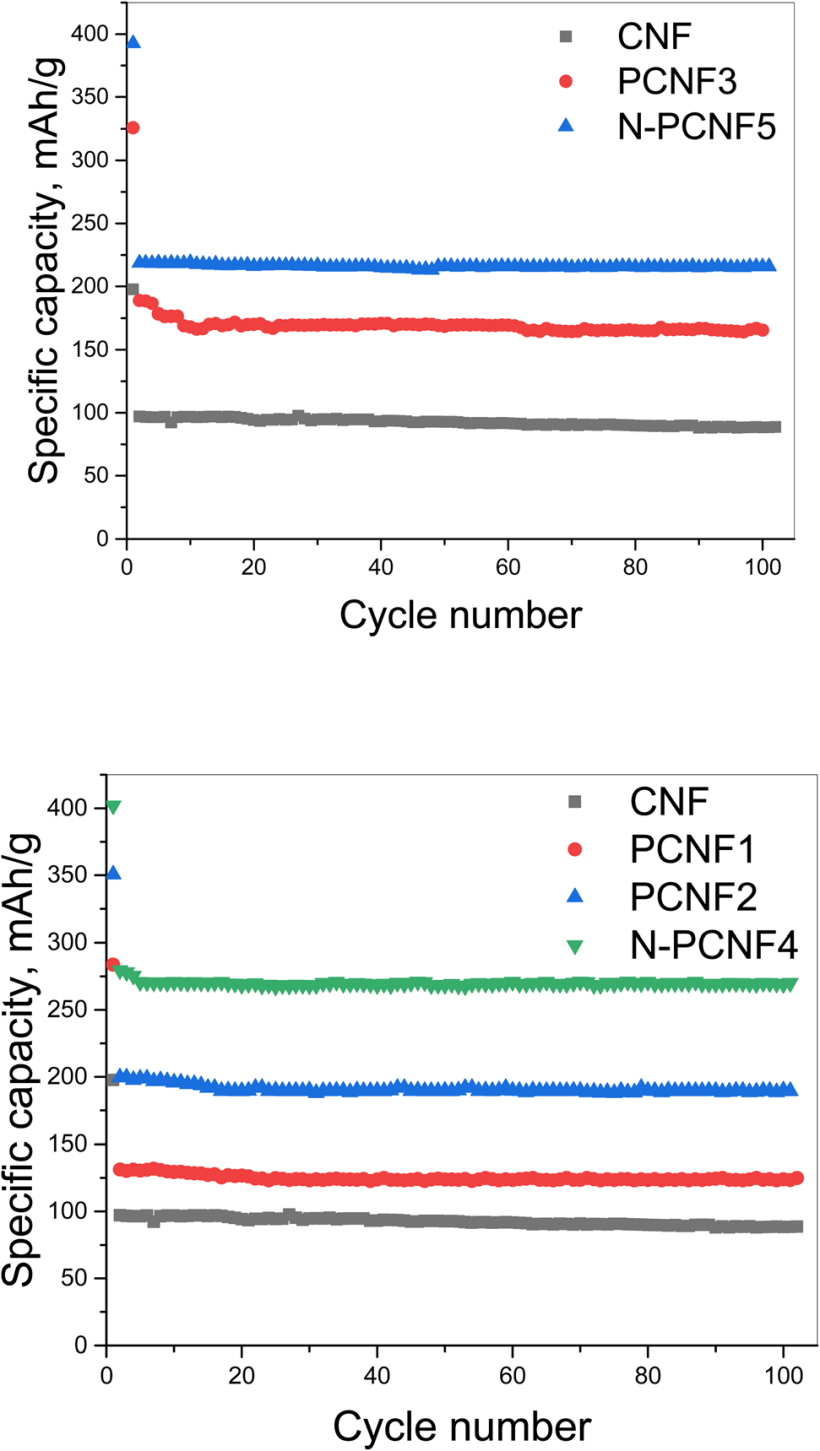
Cycling performance of CNF, PCNF1, PCNF2, PCNF3, N-PCNF4 and N-PCNF5 electrodes at a current density of 50 mA g^−1^.

The cycling performance of the electrodes at the current density of 50 mA g^−1^ was displayed in Fig. 8. The reversible capacities of around 95, 120, 190, 170, 270 and 220 mA h g^−1^ were seen from CNF, PCNF1, PCNF2, PCNF3, N-PCNF4 and N-PCNF5 respectively. The higher capacities were observed from PCNFs compared to CNF and N doping further enhanced the reversible capacities up to 270 mA h g^−1^. The highest reversible capacity of around 270 mA h g^−1^ was achieved by N-PCNF 4 electrode after 100 cycles among all studied electrodes. Enhanced capacities could be attributed to increased defects, and increased N content which well consistent with Raman and XRD results. Large amount of disordered matrix beneficial for Na^+^ storage and large content of disordered part implies larger Na^+^ storage active sites thus results in higher conductivity.^28^ Qu *et al.*^13^ also synthesized N doped carbon fiber electrodes *via* electrospinning and dopamine coating and enhanced reversible capacity of around 200 mA h g^−1^ at 50 mA h g^−1^ obtained from N doped carbon electrodes compared to that of (around 100 mA h g^−1^) carbon electrodes. Enhanced performance was attributed to porous structure and N doping. N containing functional groups promote redox reactions and defects enhance sodium ion adsorption. Moreover, porous structure contacts better with electrolyte and shorten ion pathway as well.

**Fig. 9:**
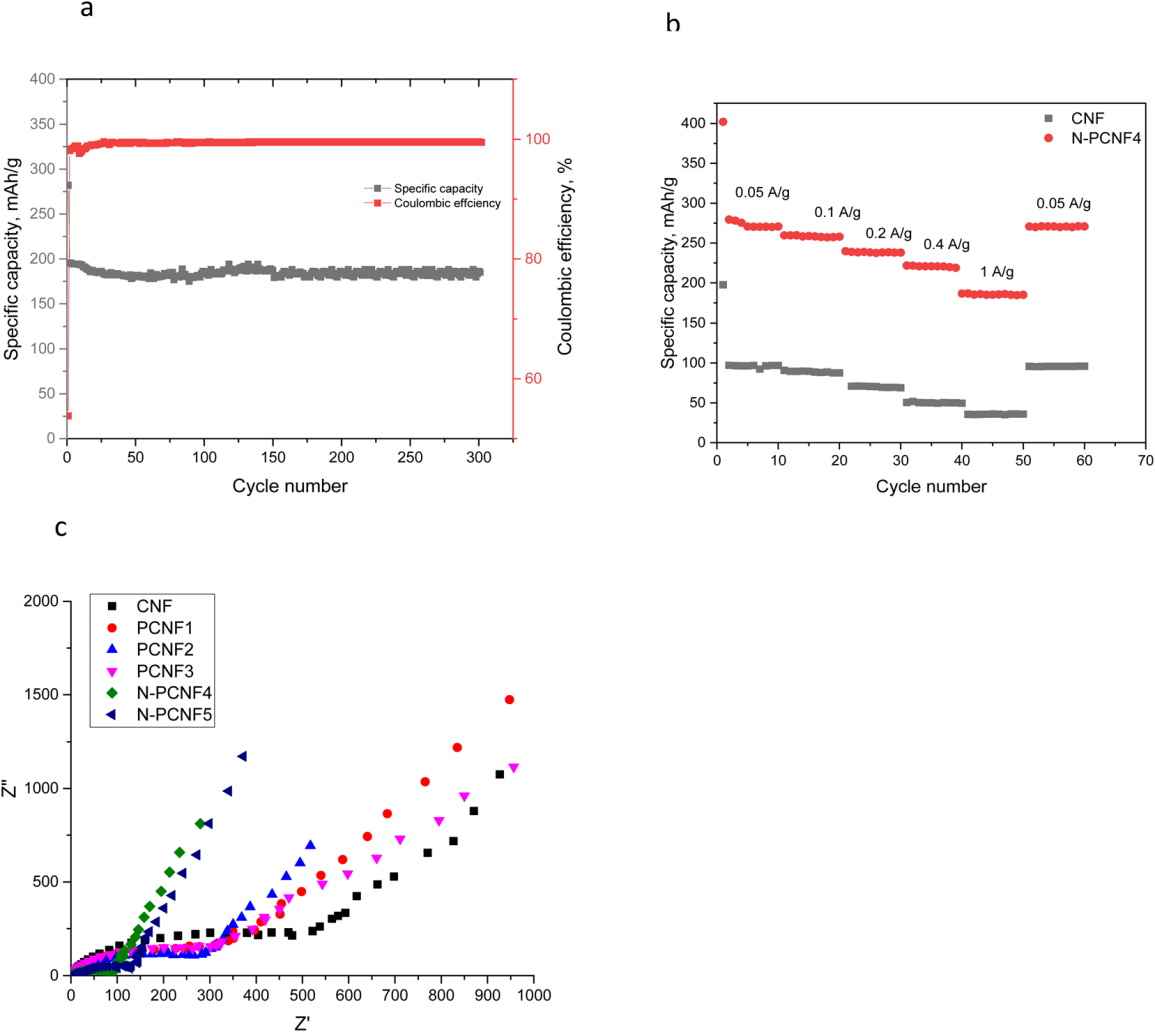
Cycling performance of N-PCNF4 electrodes at a current density of 1000 mA g^−1^ (a) C-rate performance of CNF and N-PCNF4 electrodes (b) and EIS spectra of the electrodes (c).

N doped nanoparticle on nanofiber electrode with tubular like porous structure and expanded interlayer spacings showed the highest reversible capacity among all studied electrodes. Moreover, good cycling performance of N-PCNF4 electrode at high current density of 1000 mA g^−1^ was also studied and the result was shown in Fig. 9a. At 1000 mA g^−1^, in 300 cycles reversible capacity was around 172 mA h g^−1^ with a high capacity retention over 88%. A high coulombic efficiency over 98% was also achieved. It has been reported that the doped N can enlarge the spacing distance and improve reversibility during charge discharge cycles.^29^

C-rate performance of the electrodes were investigated and the results are shown in Fig. 9b. The superior capacities of around 270, 256, 238, 220, 185 mA h g^−1^ were observed from N-PCNF 4 electrode at the current densities of 0.05, 0.1, 0.2, 0.4 and 1 A h g^−1^ whereas those of CNF electrodes were 98, 88, 69, 49 and 35 mA h g^−1^. The capacity retention exceeded 60% even after 20-fold increase in current density. Furthermore, when the C rate was back to 50 mA h g^−1^, the capacity of 270 mA h g^−1^ was observed from N-PCNF4.

The electrochemical impedance spectroscopy (EIS) spectra at an alternating current (AC) voltage of 5 mV amplitude in the 100 kHz to 0.01 Hz were also performed and presented in Fig. 9c. All of the studied electrodes showed semicircle arc shaped curves representing the charge-transfer resistance on electrode/electrolyte interface and the inclined line in the low frequency region, the Warburg impedance, is associated with the sodium-ion diffusion process in the electrodes. The diameter of semi circle is approximately 500 ohm for CNFs however that of is approximately 300 ohm for PCNFs. Moreover, the diameter decreased to 150 and 100 ohm for N-PCNF5 and N-PCNF4, respectively. Introducing porosity decreased the charge transfer resistance and N doping further decreased the resistance owing to larger accessible area and improved electronic conductivity with N inclusion which was proved by XPS survey. Lower resistance with N doping was also reported for carbon anodes and the result was attributed to enhanced conductivity.^32^

The best performance was seen from N-PCNF4 and this result could be ascribed to superior morphology that combine highly porous carbon nanofibers and PPy based N doped carbon nanoparticles. PCNFs provide 3d interconnected highly conductive electrode structure while N doped carbon nanoparticles provide high capacity along with fast diffusion kinetics. It has been reported that N doping change the electronic state of the carbon surface and thus improve electrical conductivity of the materials. Moreover, it increases defects on the surface of the carbon materials, thereby enhancing the capacity.^21^ Ghani *et al.*^7^ was also studied hard carbon based electrodes and reported superior capacity with high C rate performance. In N-PCNF4, high amount of nitrogen functional groups and porous fiber structure provided more active sites, enhanced electrical conductivity, and surface polarity, all led to excellent cycling performance. The excellent C rate performance of nitrogen doped carbon nanoparticle on highly porous nanofiber electrodes could be ascribed to novel morphology with more active sites owing to large surface area between electrodes and electrolytes, short ion diffusion distance and increased interlayer spacing which overcomes kinetic limitations. In disordered carbon electrodes, Na^+^ insert between carbon layers and Na^+^ are accommodated into mesopores. Large interlayer spacing between layers accelerates Na^+^ transport and storage. Moreover, N doping generate defects and form disordered carbon structure and thus further enhance Na^+^ absorption properties. Additional N increase electronic conductivity as well. In summary, more active sites with low charge transfer resistance, expanded interlayer spacing *via* N doping, large electrode electrolyte interface and short path for ions and electrons due to nanoparticle-on-nanofiber morphology, porous structure and N doping led to high cycling and C-rate performance.

## 4. Conclusion

Nitrogen doped carbon nanoparticle on highly porous carbon nanofiber electrodes were synthesized *via* low cost and facile approach for sodium ion batteries. Ppy coating, centrifugal spinning and heat treatment was employed to create high performance electrodes with novel nanoparticle-on-nanofiber morphology for the first time. High reversible capacity of 270 mA h g^−1^ with good cycling and C rate performance was ascribed to the highly porous, disordered carbon structure and N doping. This facile way could be employed for the development of next generation electrodes for energy storage systems.

## Conflicts of interest

There are no conflicts to declare.

## Supplementary Material

RA-013-D3RA00635B-s001

## References

[cit1] Selvamani V. (2016). *et al.*, Garlic peel derived high capacity hierarchical N-doped porous carbon anode for sodium/lithium ion cell. Electrochim. Acta.

[cit2] Qu Y. (2019). *et al.*, Novel nitrogen-doped ordered mesoporous carbon as high-performance anode material for sodium-ion batteries. J. Alloys Compd..

[cit3] Abdolrazzaghian E. (2022). *et al.*, MoS2-Decorated Graphene@ porous Carbon Nanofiber Anodes via Centrifugal Spinning. Nanomaterials.

[cit4] Lu Y. (2015). *et al.*, Centrifugally spun SnO2 microfibers composed of interconnected nanoparticles as the anode in sodium-ion batteries. ChemElectroChem.

[cit5] Chen J. (2020). *et al.*, Template-free growth of spherical vanadium disulfide nanoflowers as efficient anodes for sodium/potassium ion batteries. Mater. Des..

[cit6] Brown E. (2019). *et al.*, 3D printing of hybrid MoS2-graphene aerogels as highly porous electrode materials for sodium ion battery anodes. Mater. Des..

[cit7] Ghani U. (2022). *et al.*, Free-Standing, Self-Doped Porous Hard Carbon: Na-Ion Storage with Enhanced Initial Coulombic Efficiency. ACS Appl. Mater. Interfaces.

[cit8] Sadan M. K. (2021). *et al.*, Ultra-long cycle life of flexible Sn anode using DME electrolyte. J. Alloys Compd..

[cit9] Yu P. (2021). *et al.*, Facile construction of uniform ultramicropores in porous carbon for advanced sodium-ion battery. J. Colloid Interface Sci..

[cit10] Yanilmaz M., Kim J. J. (2022). Flexible MoS2 Anchored on Ge-Containing Carbon Nanofibers. Nanomaterials.

[cit11] Yanilmaz M. (2022). *et al.*, Centrifugally Spun PVA/PVP Based B, N, F Doped Carbon Nanofiber Electrodes for Sodium Ion Batteries. Polymers.

[cit12] Zhao L., Qu Z. (2022). Advanced flexible electrode materials and structural designs for sodium ion batteries. J. Energy Chem..

[cit13] Qu Y. (2019). *et al.*, Synthesis of nitrogen-doped porous carbon nanofibers as an anode material for high performance sodium-ion batteries. Solid State Ionics.

[cit14] Mahmud S. T. (2022). *et al.*, Recent Developments of Tin (II) Sulfide/Carbon Composites for Achieving High-Performance Lithium Ion Batteries: A Critical Review. Nanomaterials.

[cit15] Zhu H. (2018). *et al.*, Engineering capacitive contribution in nitrogen-doped carbon nanofiber films enabling high performance sodium storage. Carbon.

[cit16] Wang Y. (2018). *et al.*, Ultrastable and high-capacity carbon nanofiber anodes derived from pitch/polyacrylonitrile for flexible sodium-ion batteries. Carbon.

[cit17] Lu Y. (2015). *et al.*, Lithium-substituted sodium layered transition metal oxide fibers as cathodes for sodium-ion batteries. Energy Storage Mater..

[cit18] Lu Y. (2015). *et al.*, Centrifugal spinning: A novel approach to fabricate porous carbon fibers as binder-free electrodes for electric double-layer capacitors. J. Power Sources.

[cit19] Atıcı B., Ünlü C. H., Yanilmaz M. (2021). A review on centrifugally spun fibers and their applications. Polym. Rev..

[cit20] Atıcı B., Ünlü C. H., Yanilmaz M. (2021). A statistical analysis on the influence of process and solution properties on centrifugally spun nanofiber morphology. J. Ind. Text..

[cit21] Xu H. (2022). *et al.*, Polyimide-derived carbon nanofiber membranes as free-standing anodes for lithium-ion batteries. RSC Adv..

[cit22] Chen C. (2015). *et al.*, Use of a tin antimony alloy-filled porous carbon nanofiber composite as an anode in sodium-ion batteries. RSC Adv..

[cit23] Topuz F. (2021). *et al.*, Nanofiber engineering of microporous polyimides through electrospinning: Influence of electrospinning parameters and salt addition. Mater. Des..

[cit24] Hou T. (2017). *et al.*, Highly porous fibers prepared by centrifugal spinning. Mater. Des..

[cit25] Yanilmaz M. (2020). TiO 2-decorated porous carbon nanofiber interlayer for Li–S batteries. RSC Adv..

[cit26] Yanilmaz M., Asiri A. M., Zhang X. (2020). Centrifugally spun porous carbon microfibers as interlayer for
Li–S batteries. J. Mater. Sci..

[cit27] Huang J. (2020). *et al.*, Nitrogen-doped porous carbon derived from foam polystyrene as an anode material for lithium-ion batteries. Appl. Surf. Sci..

[cit28] Bao Y. (2018). *et al.*, Heteroatom doping and activation of carbon nanofibers enabling ultrafast and stable sodium storage. Electrochim. Acta.

[cit29] Hao M. (2018). *et al.*, Pitch-derived N-doped porous carbon nanosheets with expanded interlayer distance as high-performance sodium-ion battery anodes. Fuel Process. Technol..

[cit30] Chen L. (2022). *et al.*, Route to a Porous Carbon Nanofiber Membrane Containing Fe x C y/Fe by Facile In Situ Ion-Exchange Functionalization of the PAA Carboxyl Group: Exemplified by a Supercapacitor. ACS Appl. Energy Mater..

[cit31] Xue Y. (2019). *et al.*, Influence of beads-on-string on Na-Ion storage behavior in electrospun carbon nanofibers. Carbon.

[cit32] Kale S. B. (2021). *et al.*, Synergetic Strategy for the Fabrication of Self-Standing Distorted Carbon Nanofibers with Heteroatom Doping for Sodium-Ion Batteries. ACS Omega.

