# Correction: N-doped carbon nanoparticles on highly porous carbon nanofiber electrodes for sodium ion batteries

**DOI:** 10.1039/d5ra90019k

**Published:** 2025-02-26

**Authors:** Meltem Yanilmaz, Bülin Atıcı, Jiadeng Zhu, Ozan Toprakci, Juran Kim

**Affiliations:** a Nano-Science and Nano-Engineering Program, Graduate School of Science, Engineering and Technology, Istanbul Technical University Istanbul 34469 Turkey yanilmaz@itu.edu.tr; b Textile Engineering, Istanbul Technical University Istanbul 34469 Turkey; c Smart Devices, Brewer Science Inc. Springfield MO 65806 USA; d Department of Polymer Materials Engineering, Yalova University 77200 Yalova Turkey; e Advanced Textile R&D Department, Korea Institute of Industrial Technology (KITECH) Ansan 15588 Korea jkim0106@kitech.re.kr

## Abstract

Correction for ‘N-doped carbon nanoparticles on highly porous carbon nanofiber electrodes for sodium ion batteries’ by Meltem Yanilmaz *et al.*, *RSC Adv.*, 2023, **13**, 7834–7842, https://doi.org/10.1039/D3RA00635B.

The authors regret that the one of the affiliations (affiliation *c*) was incorrectly shown in the original manuscript. The corrected list of affiliations is as shown herein.

The Royal Society of Chemistry apologises for these errors and any consequent inconvenience to authors and readers.

