# Supplementary material for: N-Doped carbon nanoparticles on highly porous carbon nanofiber electrodes for sodium ion batteries
Source: RSC Adv. 2023 Mar 9;13(12):7834–42. doi: 10.1039/d3ra00635b (PMC9996626; doi:10.1039/d3ra00635b)
Supplement: RA-013-D3RA00635B-s001 [file RA-013-D3RA00635B-s001.pdf]

Electronic Supplementary Material (ESI) for RSC Advances.
This journal is © The Royal Society of Chemistry 2023

# N-doped carbon nanoparticles on highly porous carbon nanofiber electrodes for sodium ion batteries

**Meltem Yanilmaz[1,2]*, Bülin Atıcı[1], Jiadeng Zhu[3], Ozan Toprakci[4], Juran Kim[5]***

[1] Nano-Science and Nano-Engineering Program, Graduate School of Science, Engineering and Technology, Istanbul Technical University, Istanbul 34469, Turkey

[2] Textile Engineering, Istanbul Technical University, Istanbul 34469, Turkey

[3]Oak Ridge National Laboratory, Chemical Sciences Division, Oak Ridge, TN 37831, USA

[4]Yalova University, Department of Polymer Materials Engineering, 77200 Yalova, Turkey

[5]Advanced Textile R&D Department, Korea Institute of Industrial Technology (KITECH), Ansan 15588, Korea

**Table S1** Capacity and CE values

| Electrodes | First cycle Capacity, mAh/g | Initial Coulombic efficiency, % | Capacity retention, % |
|---|---|---|---|
| CNF | 197 | 48 | 90 |
| PCNF1 | 283 | 48 | 93 |
| PCNF2 | 350 | 54 | 94 |
| PCNF3 | 325 | 54 | 87 |
| N-PCNF4 | 402 | 66 | 97 |
| N-PCNF5 | 392 | 54 | 97 |

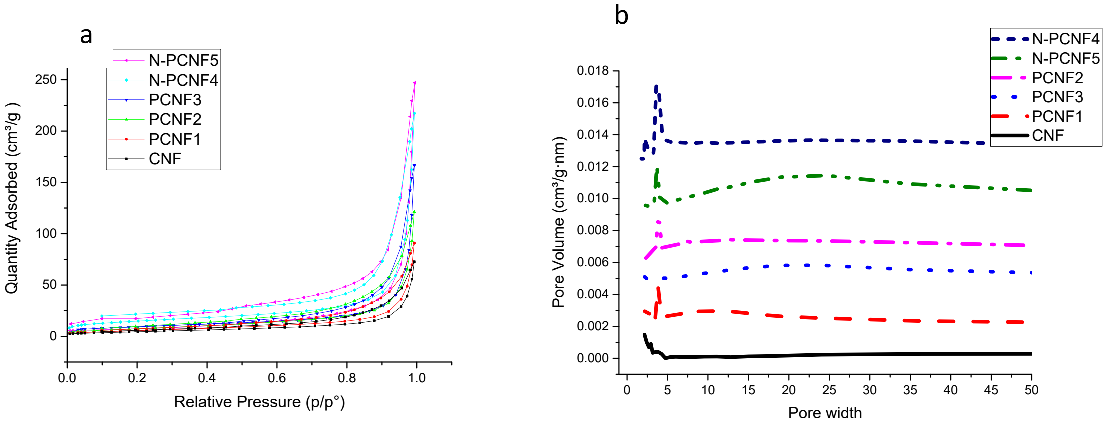


**Figure S1** $N_2$ adsorption/desorption isotherms (a), and pore size distributions (b).

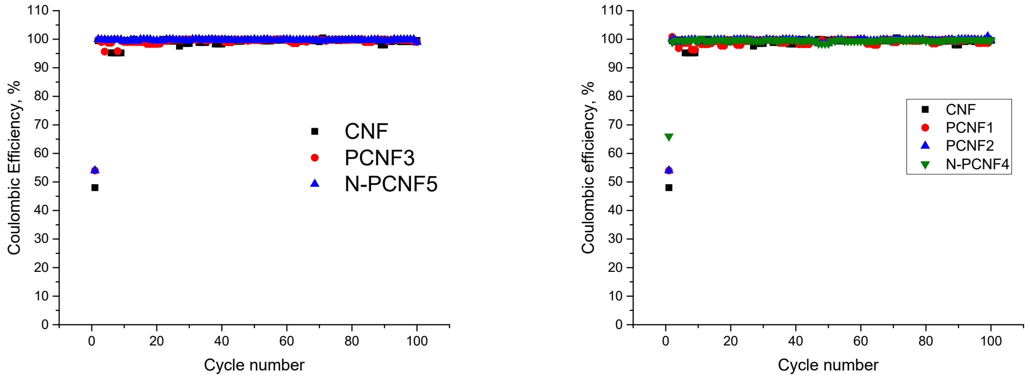


**Figure S2** Coulombic Efficiencies of of CNF, PCNF1, PCNF2, PCNF3, N-PCNF4 and N-PCNF5 electrodes.
